# Effect of Cooking Method on Vitamin C Loses and Antioxidant Activity of Indigenous Green Leafy Vegetables Consumed in Western Uganda

**DOI:** 10.1155/2022/2088034

**Published:** 2022-01-19

**Authors:** Hellen W. Kinyi, Michael Tirwomwe, Herbert Izo Ninsiima, Conrad O. Miruka

**Affiliations:** ^1^Department of Biochemistry, School of Medicine, Kabale University, Uganda; ^2^Department of Physiology, School of Medicine, Kabale University, Uganda; ^3^Department of Biochemistry, Kampala International University-Western Campus, Uganda

## Abstract

Indigenous green leafy vegetables are known to contain high levels of antioxidants and are recommended in the management of chronic diseases. However, their consumption has received lower patronage. This is partly attributed to farmers replacing the growing of indigenous green leafy vegetables with their exotic counterparts and scarce information on their nutritional benefits. This study explored the effect of cooking methods (boiling and steaming for 10 minutes) on the antioxidant activity of *Amaranthus dubius*, *Solanum nigrum*, and *Curcubita maxima.* Spectrometry was used to evaluate the reducing power and antihemolytic activity. Titration via the 2,6-indophenol method was used for measuring vitamin C and replacement titration for hydrogen peroxide scavenging activity. Cooking the vegetables by steaming or boiling caused up to 99% reduction in the vitamin C concentration of the cooked vegetables. The antioxidant activity of the cooked vegetables varied across the species assessed and cooking method used. Steaming retained and in some instances promoted the antioxidant activity of the vegetables. The study concludes that cooking by either steaming or boiling alters the antioxidant activity of indigenous vegetables.

## 1. Introduction

Organism's cells can be damaged by highly reactive atoms known as free radicals [1, 2]. Antioxidants are compounds which inhibit oxidation reactions that produce free radicals and hence protect cells [3, 4]. The imbalance between free radicals and antioxidants, known as oxidative stress, has been linked to the development of chronic health ailments such as diabetes mellitus, hypertension and other cardiovascular diseases, chronic inflammatory conditions, cancer, and neurodegenerative diseases [3, 5, 6].

Consumption of fruits and vegetables is associated with a low incidence of these oxidative-stress related diseases and aging [7]. This protective action is attributed to antioxidant vitamins such as vitamin C, *α*-tocopherol, and *β*-carotene as well as phytochemicals such as flavonoids, isoflavones, flavones, anthocyanins, catechin, and isocatechin that have shown antioxidant activity both in vitro and in vivo [2, 8, 9].

In Africa, indigenous green leafy vegetables are major ingredients of soups and sauces that accompany carbohydrate staples. They are known to contain high levels of carotenoids, vitamin C, iron, calcium, protein, flavonoids, and phenolics and hence recommended in the management of malnutrition, HIV/AIDS, diabetes, and high blood pressure [10, 11]. However, traditional vegetable species that used to be frequently consumed are being replaced with exotic species that are more expensive and less adapted to the dry/rainy seasons of the region. This is could be due to less information available on their nutritional benefits and their portrayal as food for the poor [10]. As a consequence, the average African consumes little vegetables or none at all during periods of shortages. There is therefore the need to investigate antioxidant properties of these indigenous green leafy vegetables.

In South Western Uganda, the most commonly consumed indigenous green leafy vegetables are *Amaranthus dubius*, *Solanum nigrum*, and *Curcubita maxima* [10, 11]. These vegetables are never been eaten raw but commonly cooked by either steaming or boiling [10]. However, activity of antioxidants in green leafy vegetables has been shown to be affected by cooking methods [12–14]. The aim of this study was to evaluate the effect of cooking methods on the antioxidant activity of some frequently consumed indigenous green leafy vegetables of South Western Uganda.

## 2. Materials and Methods

### 2.1. Study Design and Setting

The study was conducted at the Institute of Biomedical Research (IBR) of Kampala International University Western Campus.

### 2.2. Sample Processing and Treatment

Leafy vegetables were purchased from Ishaka Market of Bushenyi District in Western Uganda. They were purchased during the rainy season of March to May 2019, when they are readily available. They were taxonomically identified by a botanist at the Department of Biology, Mbarara University of Science and Technology. Voucher specimens were deposited and given voucher number HWK 001 *Amaranthus dubius,* HWK 002 *Curcubita maxima*, and HWK 003 *Solanum nigrum* complex.

### 2.3. Boiling and Steaming

The laboratory each of the vegetables was rinsed with distilled water, chopped to small sizes of 2-4 mm, and divided into 3 groups: A (raw), B (steamed), and C (boiled). Boiling was done as described by [15], in which, 100 g of the chopped vegetables (sample C) and 500 ml of distilled water were added to a stainless steel sauce pan. The saucepan was covered with aluminum foil to minimize vapor loss. Water was brought to boil, and the vegetables were allowed to cook for 10 minutes. The boiled leaves were dried and cooled down on a metal sieve at room temperature, placed in capped glass bottles, and refrigerated at 4°C for further analysis.

A slight modification of the method described by [16] was used to steam the vegetables. One hundred grams of the chopped vegetables (sample B) was placed in a metal sieve. The sieve was then placed on top of a stainless steel saucepan containing 500 ml of boiling distilled water and covered with aluminum foil. The vegetables were allowed to steam for 10 minutes. The steamed vegetables were cooled and stored in capped glass bottles and refrigerated at 4°C until further analysis.

### 2.4. Determination of Vitamin C Content

The amount of Vitamin C in the raw, steamed, and boiled vegetables was determined through dye titration as described in [17]. Vitamin C was extracted from 2 grams of each of the raw, steamed, and boiled vegetables, through homogenization in 100 ml of 0.5% oxalic acid. The extract was filtered using Whatman filter paper No.4, and the filtrate made up to 100 ml with 0.5% oxalic acid. Aliquots of this filtrate were titrated with freshly prepared 2,6-dichlorophenol indophenol. Ascorbic acid was used for calibration. Titrations were carried out in triplicates, and the mean of each set was used to calculate the concentration of vitamin C.

The calculation used to determine the amount of Vitamin C in the vegetable in mg/ml was

(1) $$\begin{array}{c} \text{Concentration of Vitamin C in aliquot }\left(\text{mg}\right)=\left(A‐B/S‐B\right)\times 0.05\text{mg}/\text{ml}, \end{array}$$

where *A* is the titrant volume of aliquot, *B* is the titrant volume of blank, and *S* is the titrant volume of standard.

### 2.5. Extraction for Determination of Antioxidant Activity

The raw, steamed, and boiled vegetables were extracted as described in [18] with slight modifications. The samples were dried at 25°C till a constant weight was achieved, followed by grounding using a mortar and pestle to produce a homogenous powder. Five grams of the powder was soaked in 50 ml of an aqueous-methanol solvent (70% methanol) and placed in the dark for 72 hours. The sample was then centrifuged for five minutes at 4000 g and then filtered using Whatman filter paper No. 1. The filtrate was concentrated by evaporating the methanol at 40°C over a water bath. Further drying was done in a hot air oven at 40°C. The dried extracts were collected, weighed, and stored in small bottles at 4°C and used for determination of antihemolytic activity, reducing power and hydrogen peroxide scavenging activity.

### 2.6. Determination of Reducing Power

Reducing power was determined as described in [19]. One ml of vegetable extract (1 mg/ml) at various concentrations (0.25, 0.5, 1.0, 1.5, 2.0, and 3,0 mg/ml) was mixed with 2.5 ml of phosphate buffer and 2.5 ml of 1% potassium ferricyanide, respectively, followed by incubation in a 50°C water bath for 20 minutes. After the mixture was cooled to 25°C, 2.5 ml of 10% trichloroacetic acid was added and centrifuged at 3000 rpm for 10 minutes. The supernatant (2.5 ml) was collected and mixed with 2.5 ml of distilled water and 0.5 ml of freshly prepared 0.1% ferric chloride solution. The absorbance of this mixture was measured at 700 nm. Ascorbic acid was used as the standard. All tests were done in triplicate, and the average of the three results was displayed on a graph.

### 2.7. Determination of Hydrogen Peroxide Scavenging Activity

Hydrogen peroxide radical scavenging capacity assay was measured using replacement titration as described by [20]. One ml of the vegetable extract (1 mg/ml) at various concentrations (0.25, 0.5, 1.0, 1.5, 2.0, and 3,0 mg/ml) was mixed with 3 ml of 3% ammonium molybdate, 1 ml of 0.1 mM hydrogen peroxide, 10 ml of sulphuric acid (2 M), and 7 ml of potassium iodide (1.8 M) in a conical flask. The mixture was titrated with 5.09 M sodium thiosulphate until the yellow color disappeared. All tests were performed in triplicate, and the hydrogen peroxide scavenging activity was expressed as a percentage, thus

(2) $$\begin{array}{c} \%\text{scavenging activity}=\left(\text{C}‐\text{S}/\text{C}\right)\times 100, \end{array}$$

where *C* is the average titrant volume extract of control, and *S* is the average titrant volume extract of sample.

### 2.8. Determination of Antihemolytic Activity

The inhibition of hydrogen peroxide (H_2_0_2_) induced erythrocyte hemolysis by the raw and cooked vegetables was evaluated in vitro as described by [21]. To obtain erythrocytes, 5 male wistar rats were procured and housed in the animal house of Kampala International University-Western Campus. They were provided with free access to water and food. One ml of blood was collected from the lateral tail vein of each rat, pooled together, and stored in EDTA tubes. The blood was centrifuged at 1500 rpm for 10 minutes and erythrocytes separated from the plasma and buffy coat. The erythrocytes were washed three times by centrifugation in 10 volumes of 10 mM phosphate buffered saline (PBS, pH 7.4), stored at 4°C, and used within 6 hours.

50 *μ*l of the vegetable extract at different concentrations (0.25–3 mg/ml) and 100 *μ*l of l M H_2_O_2_ (in phosphate buffered saline (PBS pH 7.4)) were added to 100 *μ*l of 5%v/v suspension of erythrocytes in PBS. The reaction mixture was shaken gently in an incubator shaker at 37°C for 3 hours, then diluted with 8 ml of PBS and centrifuged at 2000 g for 10 min. The absorbance of the resulting supernatant was measured spectrophotometrically at 540 nm. To obtain complete hemolysis, the erythrocytes were treated as above with 100 *μ*M H_2_O_2_ without the vegetable extract. The inhibitory activity of the extract was compared with that of vitamin C. Hemolysis caused by 100 *μ*M H_2_O_2_ was considered as 100%. The inhibitory concentration 50 (IC_50_) values were calculated from plots (concentration vs. % inhibition [*x*, *y*]) as the antioxidant concentration required for the inhibition of 50% hemolysis. The line of best fit was obtained from the plots and the IC50 calculated using linear regression as:

Equation for line of best fit is

(3) $$\begin{array}{c} y=ax+b,\\ \text{IC }50=\frac{\left(50-b\right)}{a}. \end{array}$$

### 2.9. Data Analysis

Shapiro-Wilk test was performed to assess the normality of data and help choose the appropriate statistical method. The Shapiro-Wilk test did not show any evidence of nonnormality and based on this, parametric tests were used to analyze the data. The overall means for reducing power, H_2_O_2_ radical scavenging capacity, and antihemolyzing activity were compared. One-way analysis of variance (ANOVA) and Tukey's multiple comparison test were used to assess the difference between raw and cooked samples. *P* value of <0.05 was regarded as significant.

### 2.10. Ethical Considerations

Approval to conduct the research was sought from Kampala International University Institutional Ethics and Research Committee. The experimental animals used in this study were treated and handled following guidelines from the National Research Council guide for the care and use of laboratory animals. The male wistar albino rats were fed on NUVITA animal feeds manufactured by Nuvita Industries Limited Kampala and provided with tap water. The animals were maintained at room temperature throughout the study period, 12 hours alternative day and night and ambient humidity. The animals were donated to the departments of Pharmacology and Biochemistry, KIUWC, for use by undergraduate students.

## 3. Results

The steamed vegetables were softer and the green color of *Amaranth* and *Solanum* intensified while that of *Curcubita* lightened compared to that of the raw vegetables. The boiled vegetables softened to the extent of breaking down and they lost the green color.

Vitamin C content was the highest in *Amaranth*s (37.80 mg/100 g), followed by *Curcubita* (35.21 mg/100 g) and least in *Solanum* (8.80 mg/100 g). Cooking by either boiling or steaming resulted in 95-99% loss of vitamin C as shown in Table 1.

**Figure 1:**
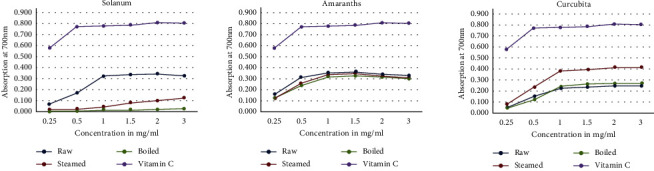
Reducing power of the raw and cooked leafy vegetables.

Cooking had varied effects on the reducing power of the leafy vegetables as shown in Figure 1. There was a statistically significant reduction (*P* = 0.0000524) in the reducing power of the steamed and boiled *Solanum*. However, alterations in reducing power of cooked *Amaranth* were not statistically significant (*P* = 0.71). Interestingly, steamed *Curcubita* had higher reducing power than the raw and boiled sample. This variation was also not statistically significant (*P* = 0.095).

**Figure 2:**
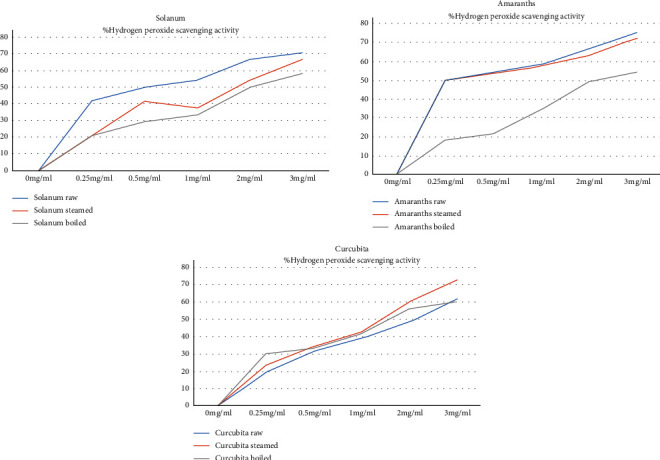
Hydrogen peroxide scavenging activity of the raw and cooked vegetables.

Boiling the *Amaranth* leaves reduced their hydrogen peroxide scavenging activity as seen in Figure 2. This reduction was statistically significant (*P* = 0.04). *Solanum* cooked by both boiling and steaming had reduced hydrogen peroxide scavenging activity. However, this reduction was not statistically significant (*P* = 0.64). Although there was no statistically significant variation in the hydrogen peroxide scavenging activity of the cooked *Curcubita*, the steamed vegetables had higher scavenging activity at concentration of 3 mg/ml.

Table 2 shows that all the vegetables were able to prevent H_2_O_2_-induced hemolysis of rat erythrocytes. However, the concentration required to cause inhibit 50% hemolysis (IC_50_) varied as the vegetables were cooked. A lower IC_50_ reflects higher antihemolytic activity.

There was statistical significant variation in the antihemolytic activity of the raw and cooked *Amaranth* (*P* = 0.0005665). Raw *Amaranth* had higher antihemolytic activity (IC_50_ of 0.56 mg/ml) compared to the steamed and boiled samples. Similarly, raw *Solanum* had the highest antihemolytic activity (IC_50_ of 0.56 mg/ml). Steamed *Curcubita* had the highest antihemolytic activity (IC_50_ 1.02 mg/ml) compared to the boiled and raw samples. This variation was not statistically significant (*P* = 0.204).

## 4. Discussion

In a bid to evaluate antioxidant activities of the three commonly consumed leafy vegetables in Western Uganda, this study ascertained that cooking affected their antioxidant activity. This correlates with other studies that show that methods used to process vegetables such as drying, shredding, steaming, blanching, boiling, sterilizing, and freezing are expected to affect the yield, composition, and activity of phytochemicals and some nutritional antioxidants such as the heat labile vitamin C [13, 22, 23]. This implies that where the vegetables are never eaten raw but are cooked by steaming or boiling, the antioxidant activity may decrease from the vegetables, resulting in a reduction of the beneficial effects.

As much as households would like to include vitamin C in their menus, this study reveals that the source as well as the method of preparation is important. Vitamin C is heat labile and water soluble and thus losses seen on cooking could be due to extremes of temperatures and leaching of the vitamin into the water used to boil the vegetables [12, 24]. Similar losses of vitamin C on cooking have been reported by [13, 25, 26].

Despite the great reduction in vitamin C levels of the steamed and boiled vegetables, they had some significant antioxidant activity. This indicates that antioxidant activity in these vegetables may be due to compounds other than vitamin C. A great number of plant secondary metabolites that have antioxidant activity have been isolated [27]. These include vitamins A and E, as well as a number of food-derived polyaromatic substances, belonging to stilbenes, flavonoids, and phenolic acids as the main classes of nutritional antioxidants [4]. A study on the evaluation of antioxidant potential in selected leafy vegetables by [28] reveals presence of phenolic compounds in uncooked methanolic and ethanolic extracts of several *Amaranth* spp. and *Curcubita maxima* leaves. Antioxidants such as tannins, flavonoids, and phenolics have also been shown in *S.nigrum* [29, 30].

This study shows that cooking has varied effects on the antioxidant capacity of the three vegetables. Different plants contain various compounds some of which are thermally labile and some are not and therefore, the same cooking method may have different effects on different types of plants. The reducing power assay assessed the ability of the vegetable extracts to reduce ferric to ferrous iron. This shows that they contain electron donors which can reduce oxidized intermediates and hence serve as primary and secondary antioxidants [1]. The low reducing power of the steamed and boiled *Amaranth and Solanum* may be due to breakdown of antioxidant compounds such as vitamins A and C, phenolics, tannins, and flavonoids and their leaching into the surrounding water [14, 31]. A study to assess the effect of steaming on the phytochemical content of *Amaranth* and *Solanum* leaves found a reduction in alkaloids, flavonoids, saponins, tannins, and phenols [16]. The increase in reducing activity seen in steamed *Curcubita* may be due to changes in plant cell wall structure, matrix modifications, and more efficient release of antioxidants during steaming and homogenization as reviewed by [32].

Hydrogen peroxide is a reactive oxygen species formed by reactions catalyzed by superoxide dismutase as part of the antioxidant cascade, xanthine oxidase, and in phagocytes [20]. It has the capacity to damage cells and macromolecules such as proteins and DNA. Though hydrogen peroxide can be reduced to water via catalase activity, excessive production could be harmful and therefore the need for other scavengers. The decrease in hydrogen peroxide scavenging activity of cooked *Amaranths* and *Solanum* extracts coupled with the fact that steaming did not cause significant variation in their hydrogen peroxide scavenging activity compared to the raw implies that the prooxidant activity may be due to peroxidase enzymes, which are inactivated by high temperatures such as boiling [33–35]. The increase in hydrogen peroxide scavenging activity exhibited by cooked *Curcubita* extracts could be explained by the fact that the leaves are harder compared to *Amaranth* and *Solanum*, and heating coupled with homogenization caused a more efficient release of antioxidants enzymes [32]. High temperatures coupled with leaching would cause the reduced activity seen in the boiled vegetables.

On considering antihemolytic activity, erythrocytes are major targets for free radicals due to the presence of both high membrane concentrations of polyunsaturated fatty acids (PUFA) and the oxygen transport associated with redox active hemoglobin molecules [36]. The antihemolytic effect is presented as the IC_50_. The half maximal inhibitory concentration (IC_50_) is a measure of the effectiveness of a compound in inhibiting a biological or biochemical function [37]. The protective effect on erythrocytes shown by the vegetables may be due to the radical scavenging activity of the bioactive components present in the vegetable extracts. Flavonoids and their glycosides are powerful antioxidants that protect erythrocytes from free radical-induced oxidative hemolysis. This is because the binding of flavonoids to the red blood cell membranes significantly inhibits lipid peroxidation, thereby enhancing their integrity [38]. Thus, in this study, inhibition of hydrogen peroxide-mediated hemolysis could indicate presence of radical scavenging polyphenols especially flavonoids as well as peroxidase enzymes. The higher antioxidant activity of the steamed *Curcubita* extracts may be due to greater release of the flavonoids due to the softening of the outer matrix by heat exposure resulting in the release of antioxidant flavonoids and peroxidase enzymes.

## 5. Conclusions

Steaming and boiling caused up to 90% loss in the vitamin C concentration of the vegetables. Boiling significantly lowered the reducing power of all the 3 vegetables. Steaming on the other hand lessened the reducing power *Amaranths and Solanum*, but increased that of *Curcubita*. Generally, steaming retained and in some instances promoted the antioxidant activity of the vegetables.

### 5.1. Limitations

The findings are limited to the effect of cooking on the antioxidant activity of the leafy vegetables. The effect of cooking on the concentration of phytochemicals with antioxidant activity (apart from vitamin C) has not been assessed. The study also assessed vegetables purchased from the market. The effect of postharvest processes such as duration and condition of storage times was not investigated.

## Figures and Tables

**Table 1:** Average vitamin C concentration in the raw and cooked vegetables in mg/100 g.

	Raw	Steamed	Boiled
Amaranths	37.8 ± 3.07	0.6 ± 0.18	0.1 ± 0.01
Curcubita	35.2 ± 5.50	0.9 ± 0.22	0.5 ± 0.21
Solanum	8.8 ± 0.52	0.5 ± 0.12	0.4 ± 0.08

**Table 2:** Antihemolytic activity of the raw and cooked vegetables.

Inhibitory concentration 50 (IC 50)			
Sample	Raw	Steam	Boil
Amaranth	0.56 mg/ml	1.55 mg/ml	1.65 mg/ml
Solanum	0.56 mg/ml	0.89 mg/ml	2.84 mg/ml
Curcubita	1.48 mg/ml	1.02 mg/ml	2.35 mg/ml
Vitamin C	0.48 mg/ml		

## Data Availability

The datasets used and/or analyzed during the current study are available from the corresponding author on request.
